# Trigger points – ultrasound and thermal findings

**Published:** 2015

**Authors:** MC Cojocaru, IM Cojocaru, VM Voiculescu, NA Cojan-Carlea, VL Dumitru, M Berteanu

**Affiliations:** * Department of Rehabilitation Medicine, Elias University Hospital, Bucharest, Romania; ** Department of Dermatology, Elias University Hospital, Bucharest, Romania; *** ”Carol Davila” University of Medicine and Pharmacy, Bucharest, Romania

**Keywords:** trigger points, myofascial pain syndrome, muscle pain, muscle ultrasound, thermography

## Abstract

**Rationale:** Muscle pain can be elicited by any irritation of the nociceptors in the muscle or central sensitization in the central nervous system. The most frequently described muscle pain syndromes are myofascial pain syndrome and fibromyalgia syndrome. Myofascial pain syndrome has a more localized manifestation, the trigger points.

**Objective:** If there is a correlation between the clinical findings, the ultrasound examination and the thermal pattern of trigger points exist.

**Material and method:** The presence of trigger points can be identified by using clinical criteria. An ultrasound examination was performed to evaluate the trigger point dimensions. The ultrasound showed an ellipsoidal hypoechogenic area in the muscle.

A thermography of the low back region was performed in order to observe the thermal pattern of the area.

**Results:** Trigger points are represented by a higher temperature area surrounded by a cooler area, probably caused by a deficit in the blood flow around those points.

**Discussion:** Infrared thermography could be a great asset for the monitoring of neuromusculoskeletal disorders and their dynamics, as well as an important aid for the initial diagnosis of conditions associated with tissue temperature alterations.

## Introduction

Muscle pain is very common in clinical practice and its most common cause is myofascial pain syndrome and fibromyalgia syndrome. Myofascial disorders are one of the most frequent pathologies for the Rehabilitation physician who plays a central role in managing the treatment [**1**]. They are also key factors that can slow down the rehabilitation process and can have a huge impact in the functional status of a patient and his activity of daily living [**2**].

The myofascial pain syndrome is defined as a regional pain syndrome caused by myofascial trigger points. The pain phenomenon is caused by the reactivation of latent trigger points [**3**]. The activation of the trigger points can be caused by a neuromusculoskeletal disorder like strain, sprain, enthesopathy, bursitis, spinal disk lesion, arthrosis etc., by poor posture, minor repetitive trauma, or by systemic diseases [**1**-**5**].

Travell and Simons described the diagnosis criteria of an active myofascial trigger point: 1) tender spots in the muscle; 2) a typical pattern of referred pain is elicited when tender spots are compressed; 3) restricted range of motion; and 4) local twitch response. The local twitch response was excluded as criterion because it would be impossible to observe it in every case [**1**,**4**,**5**].

All the matter that has a temperature greater than absolute zero degrees emits thermal radiation. Examples of thermal radiation are visible light and infrared light [**6**].

Skin temperature is conditioned by the underneath metabolic activity, skin microcirculation and vegetative nervous system activity [**7**,**8**]. Infrared thermography can be useful in monitoring the evolution of trigger points because they are associated with altered vegetative responses and abnormal microcirculation blood flow in the area surrounding the trigger point [**9**-**11**].

## Objective

This pilot study was undertaken with the aim of establishing the utility of infrared thermography in assessing monitoring trigger points by correlating clinical, ultrasound and thermal findings.

## Material and method

For this pilot study, a group of eight patients with low back pain was selected. All the patients presented a lumbar pathology that could cause spinal nerve root suffering.

**Fig. 1:**
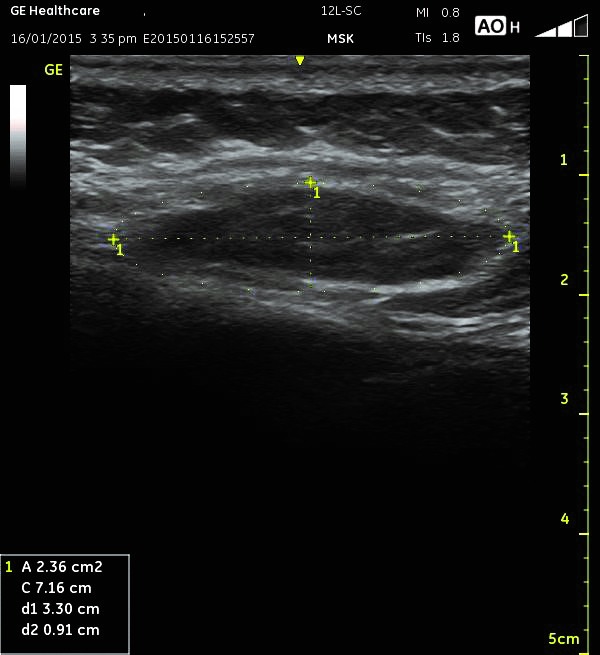
Ultrasound examination of a trigger point

The presence of trigger points was identified by using clinical criteria, and then a thermography of the low back region was performed in order to observe the thermal pattern of the area. The trigger point was evaluated by using an ultrasound examination (**Fig. 1**). Ultrasound is the last investigation because the water-based gel modifies the thermal pattern of the region. The protocol required that the examination of the patients should be performed after 30 minutes of rest without any clothes covering the lumbar region, in a room without direct sunlight or incandescent light and the temperature of the environment of 23°C. 

The thermal imaging camera used was a Trotec® EC 60 camera with a 160x120 pixels detector and a spectral range from 8 to 14 µm. 

The evaluation of the subjects was performed by a single examiner. In order to quantify the psychometric response to pain the visual analogue scale (VAS) was used.

VAS is a scale from 0 to 10, 0 representing no pain and 10 representing the worst pain ever.

Ultrasound images were acquired in B mode, with a linear transducer (General Electric 12L transducer used for soft tissue ultrasound examination) oriented in the transverse and sagittal planes.

Injection procedures were used as a treatment after the initial evaluation [**12**,**13**]. The patients were re-examined and the points were re-measured by using ultrasound, five days after the procedure.

All the patients were selected from those admitted in the Rehabilitation Department of Elias University Hospital and all of them had signed the appropriate informed consent.

## Results

The software IR-Report pro® was used for the analysis of the thermal images. Polygon selection tool was used to select the lumbar region (“Pol1”). The circle selection tool (“C” followed by a number) was used to select the trigger points. A table (**Table 1**) presents a short summary of the thermal readings from the lumbar region and from the trigger points.

Line tool (“L1”) was used to read the temperature from one end of the lumbar region, through the center of the trigger point and to the other end of the lumbar region. It was observed that the trigger point has a distinct thermal pattern: a hotter area surrounded by a cooler area. The area surrounding the trigger point was selected by using the Rectangle selection tool (R1), and the same thermal pattern was observed by using the 3D reconstruction option.

**Table 1:** Thermal readings summary

	Maximum temperature (t)	Minimum t	Average t
Patient 1			
Pol1	36.8 °C	28.6 °C	34.7 °C
C3	35.9 °C	34.5 °C	35.2 °C
C2	36.0 °C	34.7 °C	35.4 °C
C1	35.6 °C	34.5 °C	35.1 °C
Patient 2			
Pol1	33.8 °C	28.9 °C	32.0 °C
C1	33.8 °C	32.1 °C	32.9 °C
Patient 3			
Pol1	34.3 °C	31.0 °C	32.9 °C
C2	33.9 °C	32.5 °C	33.2 °C
C1	33.8 °C	32.6 °C	33.2 °C
Patient 4			
Pol1	40.2 °C	36.2 °C	38.3 °C
C1	39.6 °C	38.5 °C	39.1 °C
Patient 5			
Pol1	36.5 °C	30.2 °C	34.2 °C
C1	35.4 °C	34.1 °C	34.7 °C
Patient 6			
Pol1	36.7 °C	30.3 °C	34.4 °C
C1	35.6 °C	34.3 °C	34.8 °C
Patient 7			
Pol1	40 °C	36.1 °C	37.9 °C
C1	39.2 °C	38.1 °C	38.7 °C
Patient 8			
Pol1	36.8 °C	28.6 °C	34.7 °C
C1	36.0 °C	34.7 °C	35.4 °C

**Fig. 2:**
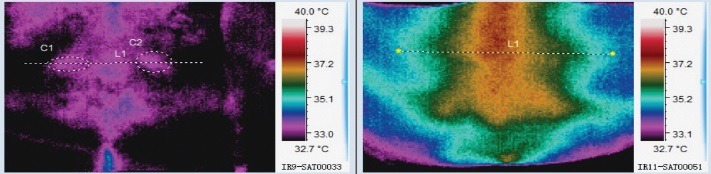
Thermal image of patient no. 2, initial and five days after the injection procedure

**Fig. 3:**
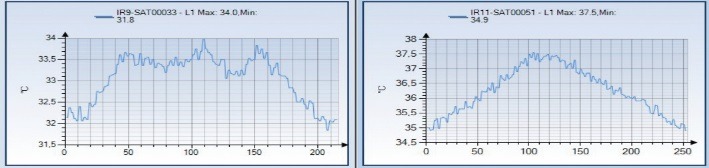
Thermal reading corresponding to L1, for patient no. 2, initial and five days after the injection procedure

**Fig. 4:**
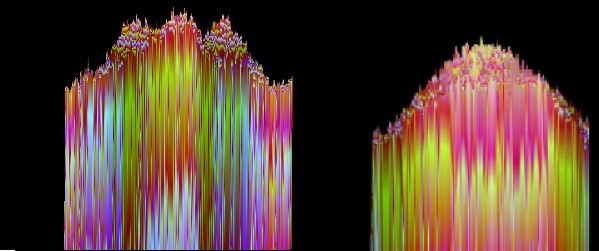
3D representation of the thermal readings corresponding to R1 region for patient no. 2, initial and five days after the injection procedure

The graphic information for the entire lot would become hard to read, so it was decided to represent only one thermography image from the initial evaluation and one from five days after the injection procedure (**Fig. 2**). These images were also analyzed by using the graphical representation of the L1 line (**Fig. 3**) and the 3D representation of the R1 area (**Fig. 4**). 

In the initial scan, it could be noticed that, both in **Fig. 3** and **Fig. 4**, there was a central spike that represented the temperature of the skin corresponding to the spine and two lateral spikes that were the thermic correspondents of the trigger points. Five days after the injection procedure, the lateral spikes were no longer present and thermal symmetry was restored.

The ultrasound showed an ellipsoidal hypoechogenic area in the muscle with an area ranging from 1,18 cm2 to 3,54 cm2.

The clinical and ultrasound examination revealed that five days after the injection procedure, the trigger points were undetectable.

VAS scale showed a decrease from an average of 6 to an average of 1, five days after the injection procedure was used as a treatment.

## Discussion

The use of infrared thermography is showing promising results in determining a thermal pattern of the trigger points and it can be a great auxiliary tool for their assessment and follow-up. Those points have a higher temperature because a muscle contraction generates heat and the cooler area is observed because there is a deficit in the blood flow surrounding these points; the exact mechanism for this phenomenon is not known but it can be caused by the metabolic disturbances in the area of a trigger point. Doppler studies revealed a deficit in the blood flow in the area of the trigger point [**14**,**15**].

As a thermal pattern, the cooler area around the trigger points can be a valuable asset for future diagnosis protocols.

Thermal imaging has limitations in medical practice. The method cannot see more than the temperature of the skin and it is unreliable as a stand-alone investigation for the diagnosis of trigger points. As an example, the same thermal pattern can be seen in a localized infection of the skin, so the clinical assessment is very important.

## Conclusion

Considering the important role of the rehabilitation physician [**16**], infrared thermography could be a great asset for the Rehabilitation Medicine Department, in monitoring neuromusculoskeletal disorders in their dynamics, as well as an important aid in the initial diagnosis of conditions associated with tissue temperature alterations. It is a fast, real time, reliable, non-invasive and non-ionizing method that can be used as a high tech auxiliary diagnostic method. High-tech and innovative technology play an important role in Rehabilitation Medicine [**17**] and thermal imaging corresponds to those criteria.

**Disclosure**

None.

**Acknowledgement**

This paper is supported by the Sectorial Operational Programme Human Resources Development (SOP HRD), financed from the European Social Fund and by the Romanian Government under the contract number POSDRU/159/1.5/S/137390.
